# Natural Products Chemistry: Advances in Synthetic, Analytical and Bioactivity Studies

**DOI:** 10.3390/molecules28145577

**Published:** 2023-07-22

**Authors:** Giovanni Ribaudo

**Affiliations:** Department of Molecular and Translational Medicine, University of Brescia, 25123 Brescia, Italy; giovanni.ribaudo@unibs.it

The chemistry of natural compounds inspired and still guides several branches of modern chemical sciences. In particular, natural compounds paved the way for the development of new therapeutic options, and the history of medicinal chemistry is rich in examples in this direction. Moreover, contemporary drug discovery tools can breathe new life into natural derivatives, as they allow the traditional uses of nature-inspired molecules to be rationalized and translated into modern medicinal chemistry. These traditional uses also allow for the identification of novel potential pathways and mechanisms of action targeted by such compounds.

The chemical diversity of scaffolds, the variety of nature and positioning of substituents, the presence of peculiar functional groups, and chirality represent the main features that enrich the intrinsic value and complexity of natural compounds. At the same time, these characteristics represent the most intriguing and challenging aspects when undertaking the study of such molecules.

The first challenge comprises the extraction process, which must be optimized to efficiently obtain the desired compound. Similarly, synthetic approaches are often very complicated when the total syntheses of natural compounds are involved. In the context of compound characterization and quantification, chemists must take into account the complications related to sample preparation and the effects of matrices. Additionally, the structural characterization of complicated natural molecules often pushes advanced analytical techniques, such as NMR and mass spectrometry, to their technical limits. A further challenge involves compound modification and the production of semi-synthetic derivatives: organic and medicinal chemists put their best efforts into the derivatization of natural molecules to produce optimized analogues, thus unleashing the potential of these semi-synthetic derivatives.

This Special Issue represents an ideal continuation and extension of the one that I previously guest-edited for the sister journal *Molbank*, entitled “Synthesis of Flavonoids and Nature-Inspired Small Molecules” [1]. The current Special Issue was launched in spring 2022 and aimed at collecting original contributions and review articles related to the extraction, structural elucidation, synthesis, analysis, and biological evaluation of natural, semisynthetic derivatives and nature-inspired molecules. Additionally, particular attention was dedicated to drug-discovery-oriented research works. This Special Issue includes contributions from researchers all over the world, testifying once again to the growing interest of the scientific community towards the applications of natural products in modern chemistry. A total of 11 research and review papers were published during this year of activity, and a brief overview of the articles is reported in the following.

More in detail, Nagata et al. described the synthesis of deuterium-labelled vitamin D analogues with applications in the field of analytical chemistry [2]. Guo et al. studied the derivatives of docosahexaenoic acid as candidates for the treatment of ovarian injury in vivo [3]. Peng et al. proposed an extract from *Musca domestica* as a tool for regulating gut microbiota [4]. Vanable et al. overviewed the state of the art of chemoenzymatic synthesis in the field of natural compounds [5]. X. Sun et al. described the application of transcriptomics and proteomics in the investigation of the anticancer mechanisms of loonamycin, a derivative of rebeccamycin, in breast cancer [6]. In the field of natural compounds of marine origin, L. Sun et al. explored the bioactive sesquiterpenoids isolated from *Aspergillus* sp. [7]. The genetic and functional aspects of glycosyltransferase *Trapa bispinosa* were the focus of the work by Ye et al. [8]. Németh-Rieder et al. proposed flavone-1,2,3-triazole hybrids as potential anticancer agents [9]. Sun and Shahrajabian overviewed the latest findings in the field of phenolic natural compounds from medicinal plants and their therapeutic applications [10]. Yin et al. reported the antimicrobial and cytotoxic activity of two alkaloids from *Arthrinium* sp. [11]. Finally, our research group contributed to this Special Issue with a comprehensive review on the role of natural and semi-synthetic compounds in ovarian cancer, in which a detailed discussion on chemical classes and involved mechanisms is provided [12].

To conclude, as the Guest Editor, I would like to thank all the authors for having chosen to publish their research in our Special Issue, as well as the Reviewers and the Assistant and Academic Editors for their support.
